# Magnification of fear and intention of avoidance in non-experienced versus experienced dental treatment in adults

**DOI:** 10.1186/s12903-021-01682-1

**Published:** 2021-07-01

**Authors:** Chia-Shu Lin, Chen-Yi Lee, Li-Ling Chen, Long-Ting Wu, Shue-Fen Yang, Tze-Fang Wang

**Affiliations:** 1 Department of Dentistry, College of Dentistry, National Yang Ming Chiao Tung University, No. 155, Sec. 2, Linong Street, Taipei, 11221 Taiwan (ROC); 2 Institute of Brain Science, National Yang Ming Chiao Tung University, Taipei, 11221 Taiwan; 3 Brain Research Center, National Yang Ming Chiao Tung University, Taipei, 11221 Taiwan; 4 Department of Oral Hygiene, College of Dental Medicine, Kaohsiung Medical University, Kaohsiung, 80708 Taiwan; 5 Department of Medical Research, Kaohsiung Medical University Hospital, Kaohsiung, 80756 Taiwan; 6 Division of Endodontics and Periodontology, Department of Stomatology, Taipei Veterans General Hospital, Taipei, 11217 Taiwan; 7 Department of Nursing, College of Nursing, National Yang Ming Chiao Tung University, Taipei, 11221 Taiwan

**Keywords:** Avoidance, Dental anxiety, Fear, Pain

## Abstract

**Background:**

Dental fear is associated with the experience of prior dental treatment and avoidance of dental visits. It remains unclear if individuals show an intention of avoidance (IA) towards treatments that they have not received (i.e., non-experienced dental treatment). The study aims to investigated (a) if individuals showed an increased fear and IA to non-experienced, compared to experienced dental treatment, and (b) if fear and IA to non-experienced treatment is associated with dental anxiety.

**Methods:**

Fear/IA of 12 common conditions of dental treatment of 402 adults were investigated. If subjects have experienced the condition, fear and IA were assessed based on subjects’ prior experience (i.e., ExpFear/ExpIA). If they have not experienced the condition, fear and IA were assessed based on their anticipation (i.e., NExpFear/NExpIA). Trait dental anxiety was assessed using the Index of Dental Anxiety and Fear (IDAF-4C+).

**Results:**

(A) NExpFear and NExpIA were significantly higher than ExpFear and ExpIA, respectively. (B) The IDAF-4C+ scores are positively correlated with NExpFear/NExpIA and negatively correlated with the magnification of fear (i.e., the discrepancy in the fear/IA of non-experienced vs. experienced conditions). (C) The condition ‘extraction of a wisdom tooth’ and ‘root canal treatment’ showed the highest ratings on NExpFear.

**Conclusions:**

Individuals may develop a high degree of fear and IA of the treatment they have not received. Trait dental anxiety plays a key role in the fear of non-experienced treatment.

## Background

Traditional views hold that one’s prior experience of dental treatment plays a key role in shaping dental fear/anxiety [1–3]. Clinical evidence supports the notion that fear of dental treatment is closely associated with previous negative treatment experience [4–7]. A recent cross-sectional study reported that the experience of past dental appointments may influence patients’ intentions of future appointments, highlighting the importance of patients' evaluation and anticipation for their intentions of dental attendance [8]. Notably, patients’ anticipation of fear and pain towards coming treatment may not reliably reflect their actual experience of treatment. Individuals may expect a stimulus to be more painful than what they actually perceived [9, 10]. Moreover, individuals may ‘overestimate’ the fear of pain of the dental treatment that they who have not experienced, compared to those they have experienced in the past [11]. Such an ‘overestimation’ of fear of pain may be associated with trait dental anxiety [11].

Critically, fear and anxiety are not only associated with pain but also avoidance of dental treatment. Around 15% to 20% of the adult population avoid or delay visiting dentists [12–14], which results in a ‘vicious cycle’ of oral health [12]. While individuals may magnify their fear of the treatment that they have not received [11], it has remained unclear if individuals also show an intention of avoidance (IA) towards the treatment that they have not experienced (i.e., non-experienced treatment). The current study aims to investigate the association between fear/IA of experienced and non-experienced conditions of dental treatment in adults.

Because dental treatment consists of a variety of procedures, which show different anxiety-stimulating effects [15], fear and IA of 12 conditions about common dental procedures were investigated in the study. Among these procedures, some of them have been widely investigated for their association with anxiety and pain during treatment, such as extraction of wisdom tooth [16–18] and endodontic treatment [9]. Notably, even for a non-invasive procedure of regular treatment (e.g., restoration and dental scaling), patients’ anxiety was markedly associated with their treatment experience. For example, in patients receiving dental scaling, increased pain was associated with increased dental anxiety [19]. In patients receiving restorative procedures, higher dental fear was associated with increased pain during treatment [20]. Here, three major hypotheses were tested:

- Hypothesis 1: Based on the previous findings of overestimation about fear of dental pain [11], it is hypothesized that fear/IA ratings are higher for the non-experienced vs. the experienced conditions of treatment.
- Hypothesis 2A: Due to the close relationship between fear and avoidance [13], it is hypothesized that one’s fear of experienced treatment is positively correlated with not only the IA of experienced treatment but also the IA of non-experienced treatment. Hypothesis 2B: Anxiety refers to a future-oriented state responding to an anticipated threat [21]. Therefore, it is hypothesized that individual scores of trait dental anxiety are positively correlated with their fear/IA of non-experienced treatment.
- Hypothesis 3: Different dental procedures have different anxiety-stimulating effects [15]. Some dental procedures may show a greater magnification factor (i.e., a greater discrepancy in the fear/IA of non-experienced vs. experienced conditions). It is hypothesized that individuals would magnify fear/IA to a lesser degree for the conditions that more people have experienced (i.e., with a higher prevalence).

## Methods

### Participants

Study samples (N = 402) were recruited independently from two sites for the current study: 201 participants from a local community recruited via advertisement and 201 dental patients from the outpatient clinic of Taipei Veterans General Hospital (Table 1). The inclusion criteria were: (a) aged between 20 and 90 years and (b) having an ability to verbally communicate with the experimenters. The exclusion criteria were: (a) having a history of major physical or psychiatric disorders and (b) feeling stressed for answering the questions (which are related to the negative experience of dental treatment). The participants provided written informed consent, approved by the Institutional Review Board of National Yang-Ming University (ID: YM106095E) and Taipei Veterans General Hospital (ID: 2018-12-003AC) before all the assessment started. In addition, to assess the test–retest reliability of the questions of dental treatment experience, 26 healthy adults were recruited to complete the questionnaire of dental treatment experience twice at a one-month interval. This group of participants also provided written informed consent, approved by the Institutional Review Board of Taipei Veterans General Hospital (ID: 2013-12-002AC).

**Table 1:** Results of the analysis of descriptive statistics across subjects

Total	402 (Male/female = 202/200)						
	Age	IDAF-4C+	HT (%)	ExpFear	ExpIA	NExpFear	NExpIA
Max	86	5.0	100	9.1	10.0	10.0	10.0
Min	20	1.0	0	1.0	1.0	1.0	1.0
Median	47	1.4	67	3.1	1.2	4.3	1.8
IQR	27	1.0	25	3.0	1.8	3.8	3.0
Mean	47	1.8	67	3.6	2.2	4.5	2.8
SD	16	0.9	20	2.0	1.8	2.4	2.4

Community	201 (Male/female = 105/96)						
	Age	IDAF-4C+	HT (%)	ExpFear^a^	ExpIA^a^	NExpFear^b^	NExpIA^b^
Max	86	5.0	100	8.9	10.0	10.0	10.0
Min	20	1.0	0	1.0	1.0	1.0	1.0
Median	46	1.4	67	3.1	1.3	4.0	2.0
IQR	30	1.0	29	2.9	1.9	3.7	3.2
Mean	45	1.8	65	3.6	2.3	4.4	2.9
SD	17	0.9	21	2.0	1.9	2.4	2.4

Clinical	201 (Male/female = 97/104)						
	Age	IDAF-4C+	HT (%)	ExpFear	ExpIA	NExpFear^b^	NExpIA^b^
Max	86	5.0	100	9.1	9.1	10.0	10.0
Min	20	1.0	8	1.0	1.0	1.0	1.0
Median	48	1.4	67	3.1	1.1	4.5	1.6
IQR	25	1.0	25	3.1	1.6	3.9	3.0
Mean	49	1.7	68	3.6	2.1	4.7	2.7
SD	16	0.9	18	2.1	1.7	2.4	2.3
Comparison^c^	n.s	n.s	n.s	n.s	n.s	n.s	n.s

*IQR* interquartile range, *n.s.* statistically not significant, *SD* standard deviation

^a^In the community sample, one subject did not receive any dental procedure listed in the study, therefore n = 200 for this variable

^b^In the community sample and the clinical sample, 13 and five subjects had received all the dental procedures listed in the study, respectively. Therefore, the variables have n = 188 and n = 196, respectively, for the community sample and the clinical sample

^c^Due to the non-normality of the distribution of all the variables, comparison between two subgroups was performed using Mann–Whitney *U* test

### Experimental procedure

#### Dental treatment experience

The questionnaire for dental treatment experience was customized with descriptions about 12 conditions of common dental procedures (Table 2), based on the previous study [22] and an earlier study [11] that adopted a set of selected dental procedures. To ensure that the conditions are common to most patients, the procedures about complicated orofacial surgery or orthodontic therapy were excluded. Trait dental anxiety was assessed using the Chinese version [18] of the Index of Dental Anxiety and Fear (IDAF-4C+) [23]. The IDAF-4C+ consists of eight questions, which assess the physiological, emotional, cognitive, and behavioral aspects of dental anxiety and fear [23]. Previous findings based on an Australian population norm revealed that the IDAF-4C+ score was associated with the avoidance of dentists and pain and anxiety related to dental visits [24]. The score was also associated with the distress related to anxiety-stimulating dental procedures, such as the postoperative pain of wisdom teeth extraction [18].

**Table 2:** Results of the analysis of descriptive statistics of each procedure

Conditions of dental treatment	Prevalence (%)	Fear		IA		Magnification factor	
		Exp	NExp	Exp	NExp	Fear (%)	IA (%)
Receiving a local anesthetic injection in the mouth	94	4.2	5.4	2.1	3.9	30	84
Having a painful tooth tapped by the dentist	85	3.7	4.3	2.2	2.7	14	23
Having a primary tooth (milk teeth) extracted in the childhood	48	4.5	4.3	3.2	3.3	− 3	2
Receiving ultrasonic scaling for removing dental calculus	92	2.5	2.5	1.7	2.1	− 1	21
A molar being drilled to remove caries	85	4.0	3.9	2.4	2.5	− 4	6
Receiving a root canal treatment	68	4.6	6.1	2.6	4.0	33	56
Having a wisdom tooth extracted by surgery	38	4.8	5.9	2.7	3.7	22	37
Feeling painful hypersensitivity when rinsing cold water	77	2.5	2.0	1.7	1.6	− 22	− 5
A caries tooth being explored with a dental instrument	91	3.8	3.9	2.2	2.5	3	13
Having the swelling gum incised and pus drained	33	4.1	5.0	2.4	2.9	22	18
Feeling excruciating postoperative pain; not being relieved even with painkillers	34	4.5	4.1	2.8	2.4	− 9	− 14
Receiving a wedge and band in between the teeth during restoration	54	2.6	3.5	1.9	2.4	35	27

For each condition of dental treatment, the participants were asked to indicate (a) whether they have experienced that condition of treatment in the past (i.e., history of treatment, HT), the degree of (b) fear of the condition (Fear), and (c) intention of avoidance of the condition (IA), respectively, according to the following instruction:

- ‘If you have previously experienced a condition about the treatment, please rate the degree of fear and intention of avoidance about that condition, by recalling your prior experience about it.’
- ‘If you have never experienced a condition about the treatment, please rate the degree of fear and intention of avoidance about that condition, by anticipating what you would feel about it, according to what you know about the treatment.’

All the ratings were scored based on a 10-point numerical rating scale, ranging from 1 (the least degree of fear/IA) to 10 (the maximal degree of fear/IA). The scores of HT, fear of experienced treatment (ExpFear), IA of experienced treatment (ExpIA), fear of non-experienced treatment (NExpFear), and IA of non-experienced treatment (NExpIA), were calculated by including the ratings from all the 12 conditions, according to the following methods:

- The conditions that subjects have experienced were indexed by the value ‘1’ and those they have not experienced were indexed by the value ‘0’. The average HT was the mean of the 12 values, denoting the proportion of conditions that a subject has experienced.
- ExpFear and ExpIA were calculated as the mean of Fear and IA, respectively, across the conditions that subjects have experienced (i.e., the conditions valued as ‘1’ for HT).
- NExpFear and NExpIA were calculated as the mean of Fear and IA, respectively, across the conditions that subjects have not experienced (i.e., the conditions valued as ‘0’ for HT).

### Statistical analysis

#### Analysis of descriptive statistics

The analysis of descriptive statistics was conducted for all the variables (age, sex, and the average of HT, IDAF-4C+, ExpFear, ExpIA, NExpFear, and NExpIA), across all subjects (n = 402) and respectively for the community and the clinical groups (n = 201 for each). Normality of the score distribution was assessed using the Shapiro–Wilk test, with *p* < 0.1 indicating non-normality.

For each of the 12 conditions of treatment, the analysis descriptive statistics was also conducted for the following variables: IDAF-4C+, ExpFear, ExpIA, NExpFear, and NExpIA. For each condition, the prevalence of experiencing a condition was calculated as the mean HT averaged across all subjects. The magnification factor is calculated as the percentage increase of fear/IA (i.e., the discrepancy between the fear/IA of non-experienced and the fear/IA of experienced conditions) normalized by the fear/IA of experienced conditions. The factor was calculated for fear and IA, respectively, as follows:

- Magnification factor of fear = 100% × (NExpFear – ExpFear)/ExpFear
- Magnification factor of IA = 100% × (NExpIA – ExpIA)/ExpIA

#### Analysis 1: comparison between experienced and non-experienced fear/IA

To test Hypothesis 1, the Wilcoxon signed-rank test was performed for comparing the scores between ExpFear and NExpFear as well as the scores between ExpIA and FearIA, across all subjects. The choice of non-parametric method is based on the non-normality of the distribution of the scores (Table 1).

#### Analysis 2: association between dental anxiety, experienced and non-experienced fear/IA

To test Hypotheses 2A, the strength of association between (a) ExpFear and ExpIA and (b) between ExpFear and NExpIA were assessed, using the Spearman’s rho coefficient. To test Hypotheses 2B, the strength of association between (a) IDAF-4C+ and NExpFear and (b) between IDAF-4C+ and NExpIA were assessed.

#### Analysis 3: association between the magnification factor of a dental condition and the prevalence of experiencing it

To test Hypothesis 3, the strength of association between (a) the prevalence of experiencing a condition and its magnification factor of fear and (b) the association between the prevalence and its magnification factor of IA were assessed, using the Spearman’s rho coefficient.

All the statistical analyses were performed using IBM SPSS Statistics (v. 24). For all the statistical tests, the level of statistical significance (alpha) was 0.05.

#### Psychometric properties of the questionnaire for dental treatment experience

For the reliability of the questionnaire, test–retest reliability was estimated according to the response from an independent group of subjects, who have completed the questionnaire at two time-points. The 26 healthy adults completed the questionnaire for dental treatment experience twice with an interval period of one month. Notably, in this version of the assessment, subjects only responded to the questions regarding their fear of dental treatment experience. The 12 conditions of dental procedures assessed are the same as those used in the main study. To evaluate the test–retest reliability of the questionnaire, Spearman’s rho coefficients were analyzed between the scores of the first and the second assessments. To evaluate the agreement between the scores, Wilcoxon signed-rank tests from a comparison between the scores from the first and the second assessments. The analyses were performed for the 12 conditions of dental procedures, respectively.

Two additional analyses were performed to assess the validity of the questionnaire. First, an analysis of discrimination validity was performed by comparing the subjects who would visit a dentist when having toothache (non-avoiders) with those who would not visit a dentist (avoiders). Based on the conclusion from previous studies [12, 24], it is hypothesized that the avoiders showed a higher ExpFear and ExpIA, compared to the avoiders. The comparison was performed using the two-tailed Mann–Whitney U test. Second, an analysis of criterion-related validity was performed to examine the association between the IDAF-4C+ score and ExpFear as well as ExpIA. Based on the conclusion from previous studies [12, 13], it is hypothesized that the correlation between the IDAF-4C+ scores and ExpFear as well as ExpIA would be both statistically significant.

#### Estimation of the sample size

The minimum sample size was calculated using the power analysis based on G*Power 3.1 [25]. As noted in the previous section, to validate the questionnaire of fear and IA of dental treatment of experience, the comparison was made between the subjects who visited a dentist and those who did not, respectively for fear and IA, using a two-tailed Mann–Whitney U test. A moderate effect size from the comparison (i.e., d = 0.45) was expected, with control of type I error and type II error at alpha = 0.05 and beta = 0.15, respectively. By these settings, the minimal number of study samples is 188. Because the study samples were recruited from two sites, 402 subjects (i.e., 201 subjects per site) were recruited, based on the calculation.

## Results

### Descriptive Analysis

The results of descriptive analyses were shown in Table 1. The proportion of sex was not significantly different (two-tailed chi-squared test with the Yates’ continuity correction, *p* > 0.05) and age, IDAF-4C+ score, ExpFear, ExpIA, NExpFear, and NExpIA were not significantly different (two-tailed Mann–Whitney U test, *p* > 0.05) between the two groups.

Table 2 revealed that the conditions “root canal treatment” and “extraction of a wisdom tooth” showed the highest scores of both ExpFear and NExpFear. The conditions “extraction of primary tooth” and “failure to relieve postoperative pain” showed the highest scores of ExpIA. In contrast, the conditions “receiving local anesthesia” and “root canal treatment” showed the highest scores of NExpIA and also the highest magnification factor of IA (Table 2).

#### Analysis 1: comparison between experienced and non-experienced fear/IA

**Fig. 1:**
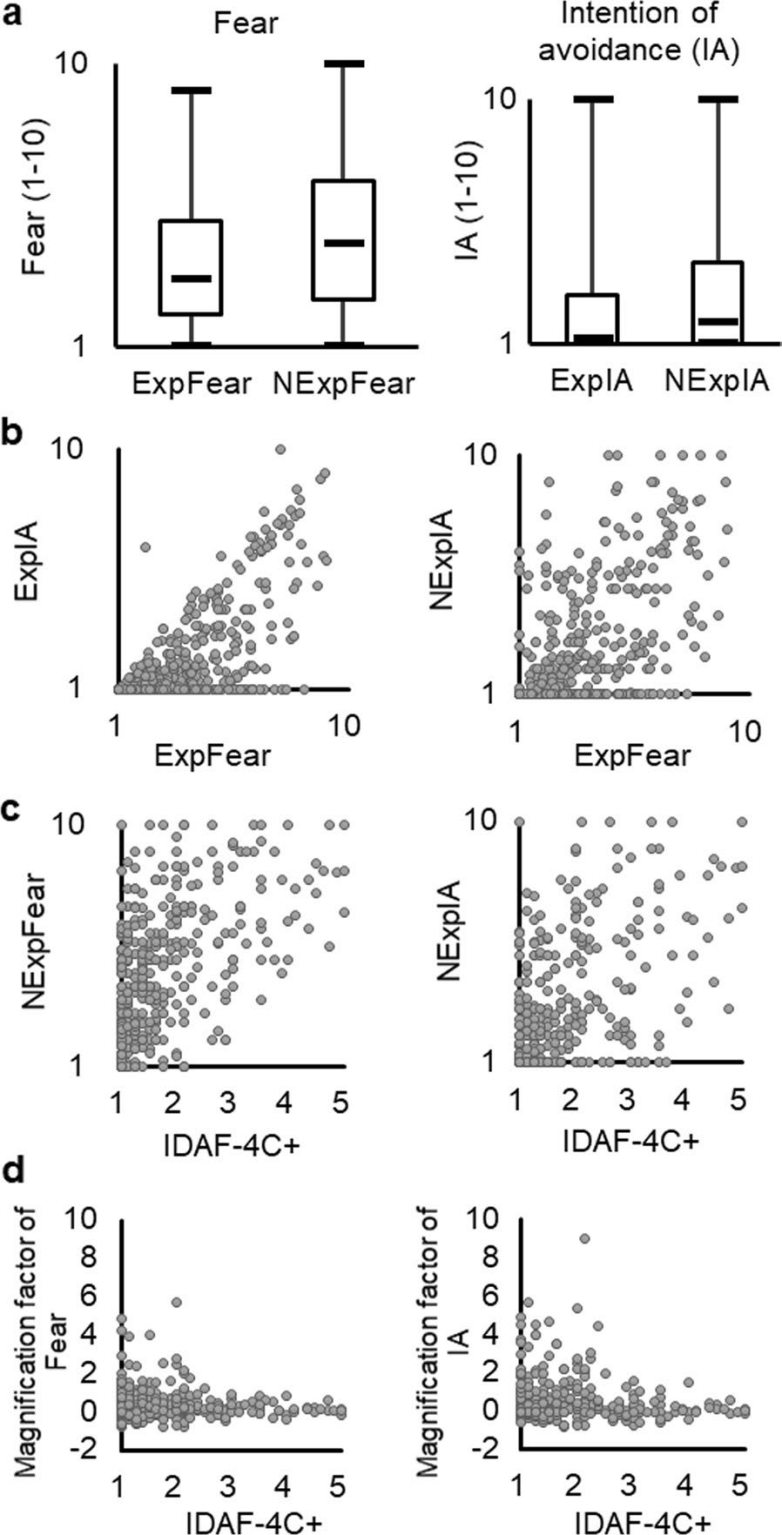
Association between experienced and non-experienced fear/intention of avoidance (IA). **a** Fear of non-experienced conditions (NExpFear) is significantly higher than fear of experienced conditions (ExpFear). IA of non-experienced conditions (NExpIA) is significantly higher than fear of experienced conditions (ExpIA). **b** ExpFear is significantly positively correlated with ExpIA and IA of non-experienced conditions (NExpIA). **c** Trait dental anxiety (IDAF-4C+ score) is significantly positively correlated with fear of non-experienced conditions (NExpFear) and NExpIA. **d** Trait dental anxiety is significantly negatively correlated with the magnification factor of fear but not that of IA

In consistent with Hypothesis 1, NExpFear was significantly higher than ExpFear (two-tailed Wilcoxon signed-rank test, *p* < 0.001) and NExpIA was significantly higher than ExpIA (two-tailed Wilcoxon signed-rank test, *p* < 0.001) (Fig. 1a).

#### Analysis 2: association between dental anxiety, experienced and non-experienced fear/IA

In consistent with Hypothesis 2, there was a positive correlation between ExpFear and ExpIA (rho = 0.62, *p* < 0.001) as well as a positive correlation between ExpFear and NExpIA (rho = 0.51, *p* < 0.001) (Fig. 1b). Additionally, there was a positive correlation between NExpFear and NExpIA (rho = 0.64, *p* < 0.001) as well as a positive correlation between ExpIA and NExpIA (rho = 0.67, *p* < 0.001). In consistent with Hypotheses 2B, there was a positive correlation between IDAF-4C+ and NExpFear (rho = 0.53, *p* < 0.001) as well as a positive correlation between IDAF-4C+ and NExpIA (rho = 0.49, *p* < 0.001) (Fig. 1c). Additionally, IDAF-4C+ was positively correlated with ExpFear (rho = 0.63, *p* < 0.001) and ExpIA (rho = 0.59, *p* < 0.001).

Additionally, the association between the discrepancy between non-experienced (NExpFear) and experienced (ExpFear) ratings, i.e., the magnification factor of fear, and dental anxiety, was assessed. The analysis showed a significant negative correlation between the magnification factor of fear and IDAF-4C+ (rho = − 0.14, *p* = 0.008) (Fig. 1d). The correlation between the magnification factor of IA and IDAF-4C+ did not show a significant result (rho = 0.05, *p* = 0.3) (Fig. 1d).

#### Analysis 3: association between the magnification factor of a dental condition and the prevalence of experiencing it

**Fig. 2:**
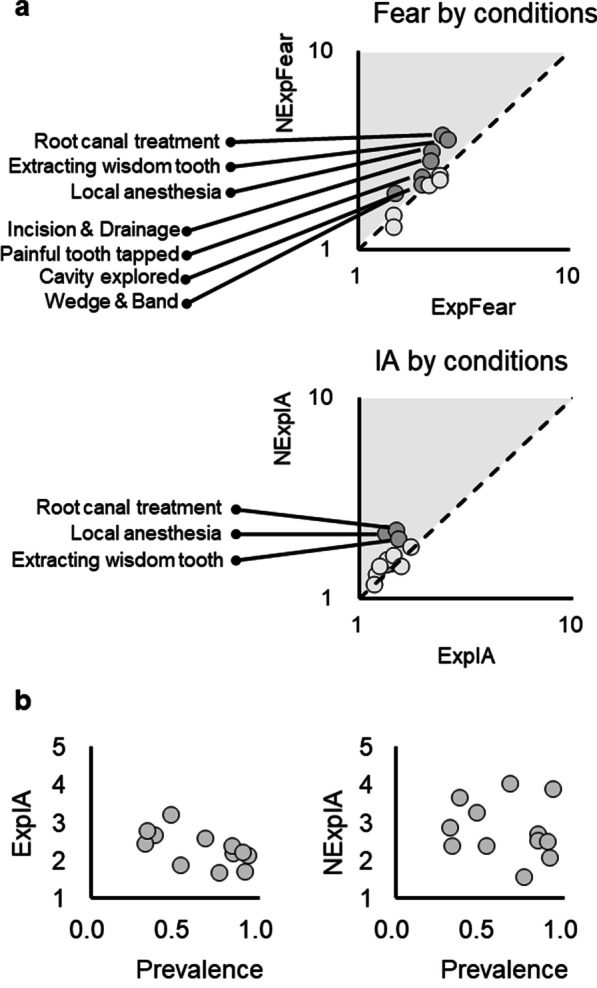
Analysis of fear and intention of avoidance (IA) by conditions of dental treatment. **a** Fear of subjects with an experience (ExpFear) and subjects without an experience (NExpFear) is significantly positively correlated across the 12 conditions. IA of subjects with an experience (ExpIA) and subjects without an experience (NExpIA) is not significantly correlated. The area with a gray shade indicates the conditions with a magnification of fear/IA in non-experienced subjects. **b** The prevalence of experiencing a condition is significantly negatively correlated with ExpIA but not NExpIA

First, the association between ExpFear and NExpFear as well as the association between ExpIA and NExpIA was assessed, across the 12 conditions. As shown in Fig. 2a, the scores of experienced and non-experienced conditions showed a significant positive correlation for fear (rho = 0.85, *p* < 0.001) but not for IA (rho = 0.54, *p* = 0.07). Critically, The correlation was not statistically significant between the prevalence and the magnification factor of fear (rho = 0, *p* = 1.0) or between the prevalence and the magnification factor of IA (rho = 0.29, *p* = 0.37). The results thus disconfirmed Hypothesis 3. However, an additional analysis revealed that the prevalence is only negatively correlated with ExpIA (rho = − 0.63, *p* = 0.027) (Fig. 2b). The correlation between the prevalence and ExpFear, NExpFear, or NExpIA, was not statistically significant.

#### Psychometric properties of the questionnaire for dental treatment experience

For the reliability of the questionnaire, 26 healthy (12 male and 14 female subjects, mean age ± standard deviation = 47.2 ± 13.7 years) participated in the assessment of test–retest reliability of the questionnaires for dental treatment experience. The interval period between the first and the second assessments is 31.5 ± 0.9 days. For the correlation between the scores from the first and the second assessments, all conditions showed a statistically significant correlation, with rho ranged from 0.89 to 0.99 (all *p* < 0.001). For the comparison between the scores from the first and the second assessments, none of the conditions showed a statistically significant difference (two-tailed Wilcoxon signed-rank test, *p* > 0.05). The findings suggested adequate reliability of the questionnaire.

For the validity of the questionnaire, the avoiders showed a significantly higher ExpFear (median = 3.7), compared to the non-avoiders (median = 3.0) (Mann–Whitney U test, *p* = 0.031). The avoiders also showed a significantly higher ExpIA (median = 2.6), compared to the non-avoiders (median = 1.1) (Mann–Whitney *U* test, *p* < 0.001). The IDAF-4C+ score was significantly correlated with ExpFear (rho = 0.63, *p* < 0.001) and ExpIA (rho = 0.59, *p* < 0.001). The findings support both discrimination validity and criterion-related validity of the questionnaire.

## Discussion

### Major findings from the current study

The current study aims to investigate the association between fear/IA of experienced and non-experienced conditions of dental treatment in adults. The major findings are:

- NExpFear was significantly higher than ExpFear. Likewise, NExpIA was significantly higher than ExpIA (Fig. 1a).
- ExpFear is positively correlated with ExpIA as well as NExpIA (Fig. 1b). The individual IDAD-4C+ scores are positively correlated with NExpFear and NExpIA (Fig. 1c). Moreover, they are negatively correlated with the magnification factor of fear (Fig. 1d).
- Among conditions of dental treatment, ‘extraction of a wisdom tooth’ and ‘root canal treatment’ showed the highest ratings on ExpFear and NExpFear (Table 2). The prevalence of experiencing a condition is negatively correlated with ExpIA (Fig. 2b).

### Association between fear and intention of avoidance

It has been widely observed that prior experience of receiving dental procedures is associated with the fear of dental treatment [4–7]. For example, patients with negative experience about dental injections would report stronger pain and anxiety during treatment [10]. Fear and pain are also associated with patients’ memory about receiving a procedure [17]. Notably, fear is considered a critical factor of avoidance of dental treatment [12–14, 26]. The findings revealed that fear may be associated with the intention of avoidance of dental treatment, even if individuals have not experienced the treatment. The findings implied that apart from prior experience, other factors may play a key role in avoidance. Apart from personal experience, social learning via language communication or observation also contributed to the development of fear [27]. Furthermore, pain can be potentiated by imagination [28] or the iconic impressions described by mass media [29].

Personal factors, such as trait dental anxiety, may play a key role in fear/IA of non-experienced conditions of treatment. Increased anxiety is associated with the response towards a future-oriented and anticipated threat [21], consistent with the condition when patients receive a procedure they have never met before. Critically, an increased trait dental anxiety, indexed by the IDAF-4C+ score, was associated with a decreased magnification factor of fear (Fig. 1d). The finding echoed the conclusion from van Wijk and Hoogstraten’s work, which reported that the ‘overestimation’ of fear of pain was reduced in the sample of highly anxious patients, relative to the non-anxious group [11]. Extending this finding, the current study reveals that some subjects with a higher trait dental anxiety still tended to rate the fear of the procedures not experienced higher than the fear of the procedures they have experienced (Fig. 1d). The findings highlight that trait dental anxiety not only reflects increased pain [30] but also signifies a greater risk for patients to fear the procedures even they have never experienced.

### Fear of dental treatment of common procedures

The current findings revealed that the experience of more invasive procedures (e.g., root canal treatment and extraction of a wisdom tooth) were fearful for both the subjects who have and have not experienced it (Table 2 and Fig. 2a). Consistently, the study by van Wijk and Hoogstraten has reported that surgical procedures (e.g., “Having a lump cut open in the mouth”, “Being drilled in the jawbone”, and “An incision in the gums”, tooth extraction, and root canal treatment, as the conditions with a high degree of overestimation in fear of pain [11]. The current results also echoed the findings from Oosterink et al., which revealed that root canal treatment and extraction of a molar as the dental experiences with the highest anxiety-provoking score (#3 and #5 in the ranking, respectively) among 67 dental stimuli [15].

By extending these findings, the current study identified a positive correlation between ExpFear and NExpFear, across the conditions (Fig. 2a). The findings may be interpreted from two aspects. First, the fear perceived by the patients who experienced a treatment (i.e., ExpFear) may be disseminated to people who have not experienced it, probably, via media or social networks, because fear can be acquired by social learning [27]. This interpretation is consistent with the findings that these two conditions are not rare to the subjects (with a prevalence of 68% and 38%, respectively) (Table 2). A second interpretation is that a negative impression (e.g., “Tooth extraction must be scaring!”) has already existed, and therefore, people have already expected a greater fear towards the treatment (i.e., a greater NExpFear) and the patients who experienced it just re-confirmed that impression (i.e., a greater ExpFear). The hypotheses regarding the cause-effect relationship between ExpFear and NExpFear may require further investigation.

### Intention of avoidance of dental treatment of common procedures

Do the fearful experience of dental treatment make people avoid receiving further dental treatment? Results from the current study have shown some critical clues regarding the fear-avoidance association. First, ExpFear was positively correlated with NExpIA (Fig. 1b), suggesting that prior experience may play a key role in the IA of the non-experienced treatment. Second, the IA scores are generally lower than the fear scores. As shown in Table 1, the median for ExpIA and NExpIA is 1.2 and 1.8, respectively, but the median for ExpFear and NExpFear is 3.1 and 4.3, respectively. The findings suggested that even though stronger fear is associated with a higher IA, in general, people are likely to attend dental treatment, evidenced by the relatively lower IA scores. In terms of the Health Belief Model [31], patients may see dentists because they perceive the symptom more severe (e.g., symptomatic pulpitis), even with a greater fear about the treatment (e.g., receiving root canal treatment). Also, the low IA may be associated with a decreased barrier of dental attendance. The approachability and availability to see a dentist in Taiwan (especially in the urban area) [32] and the lower financial burden of dental treatment due to National Health Insurance [33] may contribute to the lower IA of dental treatment.

### Limitations of the study

The results from the study need to be interpreted with several considerations from the study design. First, the dental experience questionnaire adopted only focuses on 12 conditions related to common dental procedures. Therefore, the findings cannot be generalized to the treatment associated with complicated or elective procedures, such as orthodontic, prosthodontic, or cosmetic therapies. Second, due to the non-normal distribution of scores, more sophisticated analyses (e.g., multivariable analyses) was not performed for investigating the relationship between each variable. Third, it is noteworthy that patients’ intention of attendance of dental visits is associated with positive expectations, such as getting pain relieved or improving mastication. The interplay between the negative (fear) and positive expectations would need further investigation.

### Clinical implications

Based on the novel findings presented here, three aspects for further considerations are suggested for clinical practice.

A. Individuals with a higher trait dental anxiety may tend to show the fear of the procedures that they have not experienced. Therefore, the assessment of dental anxiety will be helpful for predicting patients’ dental-visiting experience.
B. Extraction of a wisdom tooth and root canal treat present the highest fear regardless of individual prior experience. Therefore, dentists may pay attention if a negative impression about the treatment has been cast on patients, leading to their fear and avoidance.
C. Finally, recent evidence has gradually disclosed the biological mechanisms of dental fear, including its association with genetic variations [34] and brain activation [22, 35]. The biological mechanisms underlying fear/avoidance towards non-experienced stimuli would require further investigation.

## Conclusion

The novel findings suggest that individuals may develop a high degree of fear and intention to avoidance toward the conditions of dental treatment they have not experienced. Individual variations in trait dental anxiety play a key role in the fear of non-experienced treatment.

## Data Availability

The datasets generated during and analyzed during the current study are not publicly available due to regulations on the privacy of the subjects according to the guidelines from local Internal Review Board but are available from the corresponding author on reasonable request.
